# Hypermethylation of Genes Detected in Urine from Ghanaian Adults with Bladder Pathology Associated with *Schistosoma haematobium* Infection

**DOI:** 10.1371/journal.pone.0059089

**Published:** 2013-03-18

**Authors:** Xiaoli Zhong, Sumit Isharwal, Jean M. Naples, Clive Shiff, Robert W. Veltri, Chunbo Shao, Kwabena M. Bosompem, David Sidransky, Mohammad O. Hoque

**Affiliations:** 1 Department of Otolaryngology-Head and Neck Surgery, Johns Hopkins University School of Medicine, Baltimore, Maryland, United States of America; 2 Department of Molecular Microbiology and Immunology, Johns Hopkins Bloomberg School of Public Health, Baltimore, Maryland, United States of America; 3 Brady Urological Research Institute, Johns Hopkins Hospital, Baltimore, Maryland, United States of America; 4 Department of Parasitology, Noguchi Memorial Institute for Medical Research, Accra, Ghana; 5 Gono University, Savar, Dhaka, Bangladesh; 6 Department of Urology, University of Minnesota, Minneapolis, Minnesota, United States of America; Wageningen UR Livestock Research, Netherlands

## Abstract

**Purpose:**

*Schistosoma haematobium* is associated with chronic bladder damage and may subsequently induce bladder cancer in humans, thus posing a serious threat where the parasite is endemic. Here we evaluated aberrant promoter DNA methylation as a potential biomarker to detect severe bladder damage that is associated with schistosomiasis by analyzing urine specimens.

**Materials and Methods:**

A quantitative methylation-specific PCR (QMSP) assay was used to examine the methylation status of seven genes (*RASSF1A, RARβ2, RUNX3, TIMP3, MGMT, P16, ARF*) in 57 urine samples obtained from volunteers that include infected and uninfected by *S. haematobium* from an endemic region. The Fishers Exact Test and Logistic Regression analysis were used to evaluate the methylation status with bladder damage (as assessed by ultrasound examination) in subjects with *S. haematobium* infection.

**Results:**

*RASSF1A* and *TIMP3* were significant to predict severe bladder damage both in univariate (p = 0.015 and 0.023 respectively) and in multivariate (p = 0.022 and 0.032 respectively) logistic regression analysis. Area under the receiver operator characteristic curves (AUC-ROC) for *RASSF1A* and *TIMP3* to predict severe bladder damage were 67.84% and 63.73% respectively. The combined model, which used both *RASSF1A* and *TIMP3* promoter methylation, resulted in significant increase in AUC-ROC compared to that of *TIMP3* (77.55% vs. 63.73%.29; p = 0.023).

**Conclusions:**

In this pilot study, we showed that aberrant promoter methylation of *RASSF1A* and *TIMP3* are present in urine sediments of patients with severe bladder damage associated with *S. haematobium* infection and that may be used to develop non-invasive biomarker of *S. haematobium* exposure and early molecular risk assessmentof neoplastic transformation.

## Introduction

There are three species of schistosomes that infect humans, of which *Schistosoma haematobium* is the only one that affects the bladder and urogenital system. It is also an anthroponosis with no wildlife reservoir and it is confined to Africa and western Asia. As with other neglected tropical diseases, schistosome infections are widespread, affect tens of millions but have a human health impact that is poorly understood [1].This is because the infection is mainly in rural populations that are poorly served and seldom provided with post mortem examination. Previous studies in Egypt suggested that urogenital cancer was the most common cancer occurring in the country [1], [2] and due to improved preventive strategies to control *S. haematobium* infection, the incidence of bladder cancer has noticeably decreased [1], [2].

All schistosome infections are acquired through contact with infected water due to daily chores, agricultural or recreational activities. Infection with *S. haematobium* is primarily confined to the venous plexus associated with the bladder where it deposits it eggs. Eggs laid into the epithelium tend to form masses called sandy patches that induce significant inflammation. Hence, these highly antigenic pathogens form the basis for reactive tissue hyperplasia, polyps and granulomatous lesions that calcify and are the likely source of malignancy [3].

Urothelial cell carcinoma (UCC) arises from the transitional epithelium lining of the inner surface of this hollow organ and accounts for 90 percent of bladder cancer in the United States and most of the other countries. On the contrary, squamous cell carcinoma (SCC) is a distinct malignant, poorly differentiated neuroendocrine neoplasm. Although rare in the developed countries, SCC is the most common form of bladder cancer in the rural parts of Africa where *S. haematobium* is prevalent [2]. Schistosome eggs are known to secrete strongly antigenic exudates when entrapped in tissue to facilitate progress to the lumen which is associated with inflammation and in long term this chronic infection can lead to increased risk of SCC of the bladder. A variety of epidemiological studies in *S. haematobium* endemic areas point to the likely hazard of this infection but with the absence of urine biomarkers that are simple to use and reasonably effective predictors of any pathological changes including cancer. For schistosomiasis; urinary cytology, detection of parasite eggs, hematuria and detection of urine-based schistosome specific DNA are used as diagnostics [4]. Usually *S. haematobium* infections are chronic, the parasite is long lived (4–6 years), re-infections are common and associated bladder SCC start to appear early in life, even in early-mid 40’s. Although several studies have been conducted in last several decades, the extent of *S. haematobium* related public health contemporary problem remains unclear.

Molecular markers are emerging as a new option for clinical use for non-invasive early detection of chronic inflammation, precancerous lesions and cancer [5]. DNA methylation alterations, which results in chromosomal instability and silencing of tumor-related genes, are among the most common epigenetic modifications observed in human cancers and many other diseases [6], [7], [8], [9], [10], [11], [12], [13], [14].Gutierrez et al. characterized 12 cancer-related genes with methylation specific PCR in SCC and UCC from schistosome-infected cases in Egypt and their results suggested that schistosome involvement is associated with high frequency of promoter methylation in the bladder epithelium [15].

In this pilot cross sectional study, we tested the promoter methylation of a panel of infection, inflammation and cancer related hypermethylated genes in urine sediment DNA from a set of 49 cases (presence of schistosomisis infection) from Ghana with different levels of bladder damage determined by ultrasound and 8 controls (absence of schistosomiasis infection) matched for location but with no detectable bladder wall damage determined by ultrasound. Such a pilot study might provide us a basis for designing feasibility and warrant a future extension of study in a large scale molecular epidemiologic setting. As promoter methylation of all of the tested genes are related to chronic inflammation and cancer, follow-up studies will determined the value of these marker panels for molecular risk assessment of developing bladder SCC in schistosome endemic areas and will allow us to develop preventive strategies in future studies.

## Materials and Methods

### Sample Collection

The study is a continuation of an earlier project to investigate the role of *S. haematobium* parasites in causing bladder damage and co-existence of cancer biomarkers in adults sustaining chronic infection [5]. The three village sites were selected as known endemic foci of urinary schistosomiasis and the investigation was carried out in collaboration with the Noguchi Memorial Institute for Medical Research (NMIMR) and Johns Hopkins University (JHU). The study was approved by the NMIMR (IRB CPN 00203-04) and JHU (JHM IRB 03-11-12-06e) Institutional review boards. For this study, the sites were revisited by co-investigator Dr. Jean M. Naples and staff of the NMIMR in 2009 and with due publicity a number of individuals who had been previously examined were located and follow up urine specimens were taken. In all, 57 volunteers were recruited and duly informed about the study and follow up evaluation. All volunteers were signed consent forms that were retained by Dr. K. Bosompem. These volunteers were local long-time adult residents of the village who participated in the initial study. At that time they were recruited by casual interview “off the street” [5] to represent an adult population. Identities were confirmed by questionnaire after which a urine specimen was taken in a clean receptacle, numbered and placed in a cold box. These were matched with the initial ultrasound scan. If found infected, volunteers were referred to the Health Centre for further schistosomiasis examination and were treated according to the normal standard of care. The urine specimens (50–60 ml) were preserved in 3-morpholino-2-hydroxypropanesulfonic acid sodium salt (MOPSO) 90% ethanol according to the UroCor Inc. protocol [16]. These samples were then centrifuged at 4°C and the sediment from the sample was stored in the RNA/DNA Protect urine preservative (Sierra Molecular Corporation, Sonora, CA) according to manufacturer’s instructions. These samples were then stored at 4°C and finally transported to the United States in cold packs. Urine specimens were stored at −20°C until DNA was extracted. All the subjects were cancer free by ultrasound and cytology examination were done at the time of sample collection. The summary of sample information is detailed in **Table 1**.

**Table 1:** Demographic and clinico-pathological parameters of participants.

**Number of participants**	**57
**Ages**	
mean	35
Range	19–80
**Sex**	
Female	27/57
Male	30/57
* **Ultrasound Finding of S. haematobium infected subjects**	
Grade 0	14/49
Grade 1	11/49
Grade 2	9/49
Grade 3	15/49
	

* Ultrasound Finding: Please see materials and methods for details.

** 57 subjects include 49 cases and 8 controls. No bladder damage was observed in controls by ultrasound examination.

#### DNA extraction from urine

A total of 57 frozen urine cell pellets samples were washed twice with phosphate-buffered saline and incubated in 1% sodium dodecyl sulfate and 50 µg/mL proteinase K (Boehringer Mannheim, Mannheim, Germany) at 48°C overnight, followed by phenol/chloroform extraction and ethanol precipitation of DNA as previously described [16]. In general, the amount of DNA yield was about 500 ng to 2 µg per sample.

### Gene Selection

In the present study, we selected a total of seven genes to analyze their methylation status using QMSP in urine sediment. Genes were selected based on their infection, inflammation and cancer specific methylation status from previous published reports by our group and others. The genes selected were: *RASSF1A, RARβ2, RUNX3, TIMP3, MGMT, P16* and *ARF*. Aberrant methylation of *RASSF1A, RARβ2, TIMP3, MGMT, P16 and ARF* were exclusively demonstrated in UCC by our group [16]. RUNX3 methylation in UCC was reported recently [17], [18], [19], [20]. All these genes are reported to be functionally anti-proliferative.

#### Bisulfite treatment and methylation analysis

DNA extracted from urine samples was subjected to bisulfite treatment, which converts unmethylated cytosine residues to uracil residues. In brief, 500 ng to 1 µg genomic DNA from each sample was converted according to the protocol of EpiTect Bisulfite kit (Qiagen) according to the manufacturer’s instructions and stored at −80°C. Bisulfite-converted DNA was used as the template for fluorescence-based real-time PCR. Amplification reactions and calculations were performed as described previously [16]. Primers and probes were used specifically to amplify the promoters of the seven genes of interest and the promoter of a reference gene, ACTB; primer and probe sequences and annealing temperatures are provided in **Table S1**.

### Ultrasound Examination and Grading

All examinations were performed by one of us (JMN) using an Aloka SSD-500 portable ultrasound machine (Aloka, Tokyo, Japan) with 3.5 MHz curvilinear probe. Scans were recorded and scored for the following abnormalities: abnormal shape, wall irregularities/hyperplasia, masses or polyps, bladder wall calcification and hydronephrosis. All examinations were carried out according to the appropriate WHO protocol [21]. For this work four grades were used based on the following features observed during the ultrasound examination.

#### Grade 0

Normal bladder scan with smooth architecture, occasional small irregularities seen.

#### Grade 1

Abnormal shape with irregularities and areas of hyperplasia in the epithelium (not to exceed 5 mm).

#### Grade 2

As with “1” but with pronounced hyperplasia, thickening exceeding 5 mm and with occasional polyp.

#### Grade 3

As with “1” and “2” but heavy dense calcifications, nodules and polyps visible. Evidence of urethral blockage and occlusion and hydronephrosis is evident.

All individuals with *S. haematobium* infection (n = 49) were classified according to the ultrasound grade, 14 individuals with grade 0; 11 individuals grade 1; 9 individuals grade 2 and 15 individuals with grade 3. As this study was to determine the validity of biomarkers in association with severe bladder damage, only two groups were used, no severe damage (groups 0, 1 and 2) and severe bladder damage (group 3). This was done because of the small size of the sample cohort. In addition, No irregularities in bladder (i.e. grade 0) were observed by ultrasound in 8 subjects without *S. haematobium* infection.

#### Statistical analysis

Since, goal of our study is to identify methylation aberration associated with severe bladder damage associated with S. *haematobium* infection, analysis is limited to subjects with S. *haematobium* infection (i.e. n = 49). Fishers exact test was used to test for association of gene methylation status and bladder damage associated with *S. haematobium* infection. Univariate logistic regression analysis was performed to determine the independent variables significant to predict severe bladder damage associated with *S. haematobium* infection. Next, univariately significant parameters were considered in multivariate analysis. Areas under the receiver operator characteristic curves (AUC-ROC) for the ability of logistic regression models to predict severe bladder damage associated with *S. haematobium* infection were calculated.

## Results

We tested 7 gene promoters (*RSSF1A, RARβ2, RUNX3, TIMP3, MGMT, p16* and *ARF*) for methylation using urine sediment DNA extracted from 49 individuals with *S. haematobium* infection from the Densu River locations in Ghana. In this pilot study, ultrasound examinations showed severe and non-severe bladder damage in 15/49 and 34/49 respectively. Methylation of *RASSF1A, TIMP3, MGMT* were positive in 14/49, 7/49, 6/49 patients respectively. *RUNX3*, RARβ2, *p16*, and *ARF* methylation status were negative in all patients with *S. haematobium* infection. No methylation was observed in any of the tested gene and no bladder damage was noticed by ultrasound examination in all the 8 subjects without *S. haematobium* infection.

**Figure 1:**
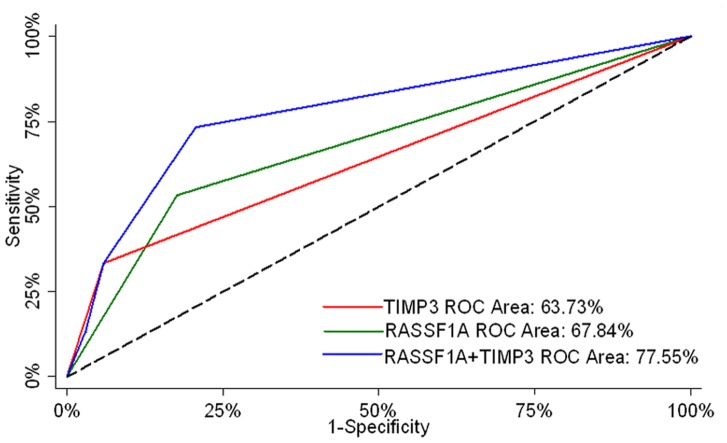
Areas under the receiver operator characteristic curves (AUC-ROC) for the ability of *RASSF1A*, *TIMP3* and combination of *RASSF1A* and*TIMP3* to predict severe bladder damage associated with *S. haematobium* infection. The AUC- ROC of *RASSF1A*, *TIMP3* and combination of *RASSF1A*and*TIMP3* were 67.84%, 63.73% and 77.55% respectively.

Among the seven genes we tested, the methylation of *RASSF1A* and *TIMP3* was significantly higher in individuals with severe bladder damage (p = 0.017 and p = 0.022 respectively) (**Table 2**). In univariate logistic regression analysis, *RASSF1A* [OR (95% CI) : 5.33 (1.39–20.45); p = 0.015] and *TIMP3* [OR (95%CI): 8.00 (1.34–47.77); p = 0.023] were significant to predict severe bladder damage (**Table 3**). However, *MGMT* methylation [OR (95%CI): 2.58 (0.46–14.62); p = 0.283] was not significantly associated to predict severe bladder damage. AUC-ROC curves for *RASSF1A* and *TIMP3* were 67.84% and 63.73% respectively (**Figure 1**).

**Table 2:** Promoter methylation status of *RASSF1A, TIMP3* and *MGMT* genes in the urine sediments of patients with *S. haematobium* infection.

Gene	Bladder Damage		p value
	Non-severe*	Severe^1^	
***RASSF1A***			
No Methylation	28	7	0.017
Methylation positive	6	8	
***TIMP3***			
No Methylation	32	10	0.022
Methylation positive	2	5	
***MGMT***			
No Methylation	31	12	0.353
Methylation positive	3	3	

* non severe = all with grade 0, grade 1 or grade 2 bladder damage.

1 severe = all with grade 3 bladder damage.

**Table 3:** Univariate and multivariate logistic regression analysis of *RASSF1A* and *TIMP3* genes.

Gene	Univariate		Multivariate	
	OR (95%CI)	p value	OR (95%CI)	p value
*RASSF1A*	5.33 (1.39–20.45)	0.015	5.35 (1.27–22.60)	0.022
*TIMP3*	8.00 (1.34–47.77)	0.023	8.05 (1.19–54.24)	0.032

When univariately significant variables were evaluated using multivariate logistic regression, both *RASSF1A* [OR (95%CI): 5.36 (1.27–22.60); p = 0.022] and *TIMP3* [OR (95%CI): 8.05 (1.19–54.24); p = 0.032] remained significant to predict severe bladder damage. The combined model, which used both *RASSF1A* and *TIMP3,* had an AUC-ROC of 77.55% (**Figure 1**). In addition, by combining *RASSF1A* and *TIMP3*, there was a significant increase in AUC-ROC compared to that of TIMP3 (77.55% vs. 63.73%; p = 0.023). However, AUC-ROC of combined *RASSF1A* and *TIMP3* model compared to that of *RASSF1A*, though showed improvement of ∼10%, was not statistically significant (77.55% vs. 67.84%; p = 0.135). This might be due to small sample size in this pilot study.

## Discussion

*S. haematobium* affects the urinary system of humans threatening the health and lives of increasing numbers of people in much of Africa and the Middle East. The infection progresses with epithelial hyperplasia and granuloma formation. Calcifications of the bladder and ureters can occur, resulting in irreversible damage of the urogenital tract that compromises lower tract urinary functions. The current dominant evidence supports that parasitic infection and resulting chronic inflammation → epithelia hyperplasia → granuloma formation → severe pathology to include calcifications that can lead to increased risk of SCC of the bladder. This is the case in particular in areas where *S. haematobium* is endemic, as opposed to the occurrence of UCC mainly in non-endemic regions [22]. Clearly, this continuous insult of repeated acute and chronic inflammation can cause DNA damage through molecules such as reactive free radical oxygen and nitrogen species producing genetic and epigenetic alterations. This can result in pre-malignant and malignant bladder changes in the infected host [23], [24], [25]. The molecular pathways the host uses to respond to such chronic infectious agents [26] and how the agents modulate this response are critical. This may ultimately determine the evolution of alterations in histological differentiation as well as an elevated risk of cancer initiation.

Development of new, non-invasive early epigenetic and other molecular and morphological biomarkers will provide an early warning system that can predict adverse changes and ultimately early intervention can save lives. The strong association between the occurrences of aberrant DNA methylation of several genesin the current urine samples with positive bladder damage determined by ultrasound findings of grade 3 pathology also suggests a potential for clinical use for early detection and prognosis follow-up in individuals who are repeatedly re-infected with schistosomiasis in endemic areas. In the current study, we have investigated DNA methylation biomarkers in urine samples from a population that has documented bladder damage but have not yet been diagnosed with bladder cancer. This population cohort was chronically infected with *S. haematobium* and exhibited varying degrees of bladder wall damage of variable extent due to infection with the schistosome parasite.

The site-specific DNA methylation markers have been shown to be associated with disease. Sequence sites have been identified where methylation correlates with cancer [27] and other diseases [28], [29]. In human gastric mucosa, the presence of *Helicobacter pylori* (HP) infection, a well-known inducer of chronic inflammation and gastric cancers is associated with high methylation levels or high incidences of methylation [30], [31], [32]. In addition, eradication of HP infection leads to a decreased incidence of CDH1 (E-cadherin) promoter methylation [32], [33]. In very recent study, using Mongolian gerbils (*Merionesunguiculatus*) model, Tohru Niwa et al [13] showed that methylation was induced specifically in gerbils with HP infection and that inflammation induced by HP infection, not HP itself, was critically involved in methylation induction. We previously reported UCC specific methylation marker and early stage UCC can be detected in urine with high sensitivity and specificity by QMSP assay [16].

Here in a pilot study, we tested the feasibility of DNA methylation based biomarkers to develop a possible screening test for high-risk populations for developing bladder pathology with severe urological clinical outcomes and a risk for developing cancer (SCC) in endemic areas where *S. haematobium* is prevalent. It is not feasible to obtain primary bladder tissues from an epidemiologically high-risk group, so we used urine sediment DNA from the individuals regularly exposed to *S. haematobium*.

Both *RASSF1A* and *TIMP3* are tumor suppressor genes, which have been found hypermethylated in bladder cancer [16] [34]. Our study showed that these two genes have high methylation frequency in schistosome-infected bladder tissues and urine samples when severe bladder damage is evident. The strong association between the occurrences of DNA methylation in the current urine samples with severe bladder damage as assessed by ultrasound also suggests a potential for clinical use of early detection and prognosis follow-up in individuals repeatedly being infected with schistosomiasis in endemic areas. We yet not know how *S.haematobium* induce methylation of a particular gene. Further biological studies needed to understand whether *S.haematobium* induces methylation in any particular site of a given gene.

Previous studies have reported the detection of genetic and epigenetic alterations in matched samples from tumor tissue and urine in patients with bladder cancer [16], [35], [36], [37], [38], [39], [40], [41], [42]. The urine DNA is presumably shed from the original primary tumor or preneoplastic lesions in the bladder wall. The promoter methylation of the studied genes has also been detected in urine of cancer patients, whereas no or less frequent methylation was detected in age-matched controls [16]. However, little is known about the importance of detection of promoter methylation of certain genes in urine of *S. haematobium*- exposed cancer-free subjects and how these epigenetic alterations are related to schistosomiasis induced cancer. In the current study, we have investigated DNA methylation biomarkers in urine samples of cancer free population. Interestingly, methylation of two genes (*RASSF1A* and *TIMP3*) is significantly associated with extensive bladder damage. We assumed that inactivation of these two genes and other still unknown genes in key molecular pathways may be altered in the individual cells with severe bladder damage and may acquire some molecularly detected transformative properties. These will eventually acquire fully transformed cells and develop frank cancer. Recently, in a nested case-control study of an extremely high-risk cohort of developing lung cancer, Belinsky et al. reported that simultaneous methylation of three or more of the six genes they tested was associated with a 6.5-fold increased risk of developing cancer [43]. Both the sensitivity and the specificity were 64% of the later study. They prospectively examined a large panel of genes for their ability to predict lung cancer and showed the promise of gene promoter hypermethylation in sputum as a molecular marker for identifying people at high risk for cancer development. Thus our findings from this study may set up a foundation for a prospective study for molecular identification of high-risk populations in areas where urinary schistosomiasis is prevalent.

This preliminary molecular epidemiologic study by an automated non-invasive approach using urine samples from *S. haematobium* infected volunteers in rural Ghanaian villages demonstrates the need to re-assess the importance of screening people in high risk rural endemic areas where schistosomiasis remains a continuing problem. Also, these new epigenetic biomarkers may prove useful to serve as early new molecular targets for bladder cancer prevention and therapeutic vaccine development. For future studies we must design a nested case control study to evaluate the best molecular and morphological biomarkers to predict early evidence of high risk pre-malignant conditions.

The advantages of screening by molecular markers are as follows: a) The urine samples from *S. haematobium* endemic area can be collected by a minimally trained health professional; b) For ultrasound testing, the individual subject needs to go to the facilities where it is available or an expert in ultrasound testing need to visit the endemic area periodically. It will be costly and infrastructure of all the rural areas in developing countries may not allow determining the risk of developing bladder SCC by ultrasound testing; c) Molecular markers can be tested in a centralized laboratory with minimal cost across the border. Like our pilot study, urine samples were collected in Ghana and we tested the markers in USA; and more importantly d) Positive molecular markers will allow us to determine cellular changes at molecular level that may not be determine by ultrasound.

### Conclusions

In this pilot study, it has been shown that severe bladder damage associated with chronic, long term infections with *S. haematobium* may be involved in epigenetic modifications which themselves may lead to increased risks of bladder cancer. If successful and validated in several data sets, this work can subsequently allow to make plan for early active treatment for the infection and reduce the morbidity and mortality due to these catastrophic bladder compromising conditions. Further, the assessment of the comparative molecular biology of UCC and SCC from the same endemic areas of Africa will permit an improved understanding of the etiopathogenesis of these two forms of bladder cancer. Finally, a larger study needs to be conducted (rural and urban) to assess the incidence of severe bladder damage and subsequent high risk to evolve into SCC and/or UCC. Few studies have attempted to identify molecular alterations due to *S. haematobium-*induced chronic infection. In order to understand the biology of *S. haematobium-*induced SCC, such epigenetic alterations could be used to determine early changes. This information may be used for targeted therapy, development of preventive strategies and non-invasive monitoring of the disease course.

## Supporting Information

Table S1 Primers and probes sequences and annealing temperatures used for QMSP.
(XLSX)
